# Potential contribution of anti-p200 autoantibodies to mucosal lesions in anti-p200 pemphigoid

**DOI:** 10.3389/fimmu.2023.1118846

**Published:** 2023-01-25

**Authors:** Yangmin Gao, Hua Qian, Takashi Hashimoto, Xiaoguang Li

**Affiliations:** ^1^ Dermatology Hospital of Jiangxi Province, Jiangxi Provincial Clinical Research Center for Skin Diseases, Candidate Branch of National Clinical Research Center for Skin Diseases, Dermatology Institute of Jiangxi Province, The Affiliated Dermatology Hospital of Nanchang University, Nanchang, China; ^2^ Department of Laboratory Medicine, Chronic Disease Research Center, Medical College, Dalian University, Dalian, China; ^3^ Department of Dermatology, Osaka Metropolitan University Graduate School of Medicine, Osaka, Japan

**Keywords:** anti-p200 mucous membrane pemphigoid, anti-p200 pemphigoid, mucosal lesions, oral mucosa, genital mucosa

## Abstract

Anti-p200 pemphigoid is a relatively rare subepidermal autoimmune bullous disease (AIBD), which was firstly reported by Detlef Zillikens, Takashi Hashimoto and others in 1996. Skin lesions are considered as the major clinical features of this disease, with occasional involvement of mucosal lesions. The mechanism of mucosal lesions involved in anti-p200 pemphigoid is still unclear. In the present study, we aimed to analyze published data on cases and case series of anti-p200 pemphigoid with mucosal lesions and explored the potential contribution of anti-p200 autoantibodies to mucosal lesions. A total of 32 papers that comprised 52 anti-p200 pemphigoid patients with various mucosal lesions were included in this review. Oral lesions were involved in 75.0% patients, followed by genital lesions (26.9%) and ocular lesions (11.54%). Only one patient had psoriasis, 26.9% patients had multiple mucosal lesions, and 30.8% cases had comorbidity of other AIBDs, particularly anti-laminin (LM) 332-type mucous membrane pemphigoid (MMP). In comparison with anti-LM332-type MMP, anti-BP180-type MMP and epidermolysis bullosa acquisita, higher frequency of genital lesions was identified as a unique character of anti-p200 pemphigoid with mucosal lesions. These results indicated that anti-p200 autoantibodies might contribute to mucosal lesions in a pattern different from other MMP-related autoantibodies, although its pathogenetic mechanisms are still unclear.

## Introduction

Subepidermal autoimmune bullous diseases (AIBDs) are a group of rare autoimmune skin diseases characterized by autoantibodies against epidermal basement membrane zone (BMZ) proteins, which include bullous pemphigoid (BP), mucous membrane pemphigoid (MMP), anti-p200 pemphigoid, epidermolysis bullosa acquisita (EBA) and others (1). The anti-BMZ autoantibodies disrupt the epidermal cell adhesive function and anchoring mechanism within epidermal-dermal junction, leading to the separation of skin and/or mucous membranes and the formation of blisters and/or erosions.

Anti-p200 pemphigoid was first reported in 1996 in two papers by Detlef Zillikens, Hashimoto and others and by Chen, Hashimoto and others, as a novel AIBD disease entity with IgG autoantibodies against a 200-kDa dermal protein (p200) (2, 3). Then, Kawahara, Detlef Zillikens, Hashimoto and others showed that p200 is localized in the lower lamina lucida of the epidermal BMZ (4). In 2009, Dainichi, Hashimoto and others reported that p200 is possibly LMγ1 (5, 6), although there might be other candidate molecules as p200.

Anti-p200 pemphigoid presents mainly skin lesions and occasionally mucosal lesions. The clinical presentation of anti-p200 pemphigoid is polymorphic, and may mimic to those of other subepidermal AIBDs, such as BP and EBA (7). A number of studies reported that anti-p200 pemphigoid is often associated with psoriasis (8). Histopathologically, lesional skin biopsy specimen shows subepidermal blister with prominent inflammatory infiltrations of neutrophils, followed by lymphocytes and eosinophils (4).

Diagnosis of anti-p200 pemphigoid is based on clinical, histopathological and immunological findings, particularly the presence of anti-p200 autoantibodies. In anti-p200 pemphigoid, direct immunofluorescence (DIF) using patient perilesional skin/mucosa usually shows linear deposits of IgG and C3 to the BMZ (9–13). Indirect immunofluorescence (IIF) using normal human skin detects circulating IgG antibodies to the BMZ (14–17), which reacts with dermal side of 1M NaCl-split normal human skin (ssIIF) (14, 18–21). Immunoblotting (IB) using normal human dermal extract detects IgG reactivity with p200 (14, 22–25).

In some cases of anti-p200 pemphigoid, additional autoantibodies to LM332, BP180, type VII collagen and BP230 were also detected (10, 22, 26–28).

Although autoantibodies in anti-p200 pemphigoid sera are considered to be pathogenic, their pathogenicity is not yet fully elucidated (29, 30).

MMP is a rare and chronic mucosal-dominant subepidermal AIBD. Known autoantigens of MMP include BP180, BP230, LM332, integrin α6β4 and type VII collagen (31). Recently, our group presented a unique AIBD case with only oral mucosal lesion, which was positive for p200 but negative for all known MMP-related autoantigens. We considered that the anti-p200 autoantibodies might cause the mucosal lesions, and suggested the new MMP subtype, anti-p200 MMP (14).

In the present study, we summarized the clinical, histopathological and immunological features of anti-p200 pemphigoid presented with mucosal lesions published either as sporadic case reports or in case series, for the better understanding of the potential contribution of anti-p200 autoantibodies to development of mucosal lesions.

## Materials and methods

### Literature review and article selection

A literature review was performed on PubMed (1996–2022) for anti-p200 pemphigoid cases by using the terms, p200, laminin gamma 1, lamγ1, LMγ1, anti-p200, anti-laminin gamma 1, anti-lamγ1, anti-LMγ1, anti-p200 pemphigoid, anti-laminin gamma 1 pemphigoid, anti-lamγ1 pemphigoid, anti-LMγ1 pemphigoid. Articles published in English were considered for eligibility. We also screened the references of the included studies for additional eligible publications. Then, a literature review on anti-p200 pemphigoid was conducted for several criteria between the cases with mucosal lesions and the cases only with skin lesions by statistical evaluation.

### Selection criteria

All the studies reporting one or more cases with a diagnosis of anti-p200 pemphigoid were included. Criteria for the diagnosis were listed below:

- clinical manifestations of subepidermal AIBD.
- linear deposition along the BMZ on DIF or showing positive signal along the dermal side of the split skin on ssIIF.
- positive signals for p200/LMγ1 by IB using human dermal extract or recombinant proteins of LMγ1.
- exclusion of other subepidermal AIBDs.

### Data extraction

Each screened article was critically reviewed. The following variables were gathered as available: age, sex, location, final diagnosis, clinical features, histopathology, immunological profiles and associated comorbidities. According to the autoantigens detected, we classified the patients into two subgroups, “sole p200”, which reacted only with p200, and “multiple antigens (Ags)”, which reacted with p200 and other antigen(s).

### Data analyses

The clinical, histopathological and immunological features of anti-p200 pemphigoid with mucosal lesions were summarized, and compared with anti-p200 pemphigoid only with skin lesions, other MMPs and EBA. The statistical analyses were performed using chi-square test, fisher exact test by SPSS software, version 23 (SPSS, Chicago, IL, USA). The *p* values less than 0.05 were considered as statistically significant.

## Results

### Demographic characteristics and diagnosis of the patients

From the literature, we selected total of 154 anti-p200 pemphigoid patients, among whom, 52 (33.8%) patients presented with mucosal lesions. The demographic characteristics of these 52 patients were summarized in **Table 1**. These 52 patients, reported from eight countries, were composed of 30 males (age, mean ± SD, 63.7 ± 16.0), 19 females (58.7 ± 21.8) and 3 with no gender information. There are no statistical differences for ages of disease onset among different countries (all *p*>0.05). In these 52 cases, 38 (73.1%) were diagnosed as exclusively anti-p200 pemphigoid, while 14 (26.9%) were diagnosed as anti-p200 pemphigoid concurrent with other AIBDs or other autoantibodies, and 9 of the 14 patients were associated with anti-LM332-type MMP.

**Table 1:** Demographic characteristics of the 52 cases of anti-p200 pemphigoid with mucosal lesions.

Items	Mean of ages (SD)	Number of cases (Percentage)
Gender		
Male	63.7 (16.0)	30 (57.7%)
Female	58.7 (21.8)	19 (36.5%)
Unknown	Unknown	3 (5.8%)
Geographical distribution of reported cases		
Germany	56.1 (21.7)	14 (26.9%)
France	75.1 (12.3)	11 (21.2%)
Japan	59.5 (16.8)	10 (19.2%)
India	58.2 (22.5)	9 (17.3%)
USA	65.7 (15.3)	3 (5.8%)
China	55.7 (7.0)	3 (5.8%)
Korea	49 (/)	1 (1.9%)
Poland	52 (/)	1 (1.9%)
Final diagnosis		
Anti-p200 pemphigoid	60.9 (20.1)	38 (73.1%)
Anti-p200 pemphigoid + anti-LM332-type MMP	63.2 (5.8)	8 (15.4%)
Anti-p200 pemphigoid + EBA	58.5 (9.2)	2 (3.8%)
Anti-p200 pemphigoid + anti-BP180-type MMP	87 (/)	1 (1.9%)
Anti-p200 pemphigoid + BP	68 (/)	1 (1.9%)
Anti-p200 pemphigoid + BP + anti-LM332-type MMP + anti-CNTN1-positive IP	77 (/)	1 (1.9%)
Anti-p200 MMP	49 (/)	1 (1.9%)

LM332, laminin 332; MMP, mucous membrane pemphigoid; BP, bullous pemphigoid; EBA, epidermolysis bullosa acquisita; anti-CNTN1-positive IP, anti-contactin-1-positive inflammatory polyneuropathy; “/”, not applicable.

### The clinical, histopathological and immunological features of the 52 cases of anti-p200 pemphigoid with mucosal lesions

Clinically, 52 cases had mucosal lesions on the oral (75.0%), genital (26.9%), ocular (11.5%), nasal (9.6%), pharyngeal (1.9%) and esophageal (1.9%) mucosae (**Table 2**). Fourteen (26.9%) cases showed lesions on multiple mucosal sites, including 3 cases on 4 sites, 2 cases on 3 sites and 9 cases on 2 sites (**Table 3**). In these 14 cases with multiple mucosal lesions, oral, genital, ocular, nasal lesions were found in 14, 10, 5 and 5 cases, respectively (**Table 3**).

**Table 2:** Clinical, histopathological and immunological features of the 52 cases of anti-p200 pemphigoid with mucosal lesions.

Item			Sole p200 Ag-M (36) *	Multiple Ags-M (16) *	All cases (52)
**Mucosal lesions**	Oral	Erosion	9 in 36 (25.0%)	8 in 16 (50.0%)	17 in 52 (32.7%)
		Erythema	3 in 36 (8.3%)	0	3 in 52 (5.8%)
		Blister	5 in 36 (13.9%)	9 in 16 (56.3%)	14 in 52 (26.9%)
		Ulceration	1 in 36 (2.8%)	1 in 16 (6.3%)	2 in 52 (3.8%)
		Unknown	16 in 36 (44.4%)	2 in 16 (12.5%)	18 in 52 (34.6%)
		Total	27 in 36 (75.0%)	12 in 16 (75.0%)	39 in 52 (75%)
	Ocular	Hyperaemia	1 in 36 (2.8%)	0	1 in 52 (1.9%)
		Erosion	1 in 36 (2.8%)	1 in 16 (6.3%)	2 in 52 (3.8%)
		Blister	0	1 in 16 (6.3%)	1 in 52 (1.9%)
		Ulceration	0	1 in 16 (6.3%)	1 in 52 (1.9%)
		Scar	0	0	0
		Unknown	2 in 36 (5.6%)	1 in 16 (6.3%)	3 in 52 (5.8%)
		Total	3 in 36 (8.3%)	3 in 16 (18.8%)	6 in 52 (11.5%)
	Genital		8 in 36 (22.2%)	6 in 16 (37.5%)	14 in 52 (26.9%)
	Nasal		1 in 36 (2.8%)	4 in 16 (25.0%)	5 in 52 (9.6%)
	Esophageal		0	1 in 16 (6.3%)	1 in 52 (1.9%)
	Pharyngeal		0	1 in 16 (6.3%)	1 in 52 (1.9%)
	Laryngeal		0	0	0
	Unknown		5 in 36 (13.9%)	3 in 16 (18.8%)	8 in 52 (15.4%)
	Total		36 in 36 (100.0%)	16 in 16 (100.0%)	52 in 52 (100%)
**Skin lesions**			35 in 36 (97.2%)	16 in 16 (100.0%)	51 in 52 (98.1%)
**Psoriasis**			0	1 in 16 (6.3%)	1 in 52 (1.9%)
**Malignancy**	Metastatic ovarian carcinoma		0	1 in 16 (6.3%)	1 in 52 (1.9%)
	Metastatic esophageal cancer		1 in 36 (2.8%)	0	1 in 52 (1.9%)
	Total		1 in 36 (2.8%)	1 in 16 (6.3%)	2 in 52 (3.8%)
**Histopathology feature**	Subepidermal blistering		35 in 36 (97.2%)	13 in 13 (100.0%)	48 in 49 (98%)
	Neutrophils infiltration		15 in 36 (41.7%)	8 in 13 (61.5%)	23 in 49 (46.9%)
	Eosinophils infiltration		9 in 36 (25.0%)	10 in 13 (76.9%)	19 in 49 (38.8%)
	Lymphocytes infiltration		4 in 36 (11.1%)	5 in 13 (38.5%)	9 in 49 (18.4%)
**Detection methods**	DIF for BMZ (IgG)		23 in 24 (95.8%)	11 in 12 (91.7%)	34 in 36 (94.4%)
	DIF for BMZ (IgA)		5 in 7 (71.4%)	1 in 5 (20.0%)	6 in 12 (50%)
	DIF for BMZ (IgM)		0	1 in 5 (20.0%)	1 in 7 (14.3%)
	DIF for BMZ (C3)		23 in 24 (95.8%)	11 in 11 (100.0%)	34 in 35 (97.1%)
	IIF for BMZ (IgG)		4 in 5 (80.0%)	4 in 5 (80.0%)	8 in 10 (80.0%)
	IIF for BMZ (IgA)		1 in 1 (100.0%)	0	1 in 2 (50.0%)
	ssIIF reactive with both epidermal and dermal side (IgG)		0	4 in 4 (100.0%)	4 in 4 (100.0%)
	ssIIF reactive with dermal side (IgG)		26 in 27 (96.3%)	9 in 9 (100.0%)	35 in 36 (97.2%)
	ssIIF reactive with dermal side (IgA)		4 in 5 (80.0%)	1 in 1 (100.0%)	5 in 6 (83.3%)
	IB of normal human epidermal extracts for p200 (IgG)		6 in 9 (66.7%)	0	6 in 9 (66.7%)
	IB of normal human dermal extract for p200 (IgG)		29 in 30 (96.7%)	15 in 15 (100.0%)	44 in 45 (97.8%)
	IB of normal human dermal extract for p200 (IgA)		1 in 1 (100.0%)	0	1 in 1 (100.0%)
	IB of LM521 RP for LMγ1 (IgG)		1 in 1 (100.0%)	1 in 1 (100.0%)	2 in 2 (100.0%)
	IB of LM111 RP for LMγ1 (IgG)		1 in 1 (100.0%)	1 in 1 (100.0%)	2 in 2 (100.0%)
	IB of LM411 RP for LMγ1 (IgG)		0	1 in 1 (100.0%)	1 in 1 (100.0%)
	IB of C-terminus of LMγ1 RP for LMγ1 (IgG)		9 in 11 (81.8%)	1 in 1 (100.0%)	10 in 12 (83.3%)
**Combined with other antigens**	LM332	α3 subunit	/	2 in 16 (12.5%)	2 in 52 (3.8%)
		β3 subunit	/	3 in 16 (18.8%)	3 in 52 (5.8%)
		γ2 subunit	/	4 in 16 (25.0%)	4 in 52 (7.7%)
		Unknown	/	3 in 16 (18.8%)	3 in 52 (5.8%)
		Total	/	9 in 16 (56.3%)	9 in 52 (17.3%)
	BP180		/	6 in 16 (37.5%)	6 in 52 (11.5%)
	Type VII collagen		/	2 in 16 (12.5%)	2 in 52 (3.8%)
	BP230		/	1 in 16 (6.3%)	1 in 52 (1.9%)
	Total#		/	16 in 16 (100.0%)	16 in 52 (30.8%)

LM332, laminin 332; Ag, antigen; *all 52 cases of anti-p200 pemphigoid with mucosal lesions were classified into 2 subgroups, (i) those with only p200 as autoantigen (sole p200 Ag-M subgroup) and (ii) those with p200 plus other autoantigens (multiple Ags-M subgroup); DIF, direct immunofluorescence; BMZ, basement membrane zone; IIF, indirect immunofluorescence; ssIIF, IIF using 1M NaCl-split skin; IB, immunoblotting; RP, recombinant protein; #, the total numbers of patients who combined with other antigens; “/”, not applicable.

**Table 3:** Mucosal lesions distribution of 14 anti-p200 pemphigoid patients with multiple sites of mucosal lesions.

Case no.	Oral	Ocular	Genital	Nasal	Esophageal	Pharyngeal	Laryngeal	Total numbers of affected mucosae	References
**1**	+	+	+	+	–	–	–	4	Li et al., 2014 (32)
**2**	+	+	+	+	–	–	–	4	Mitsuya et al., 2008 (33)
**3**	+	–	+	+	–	+	–	4	Sarrazin et al., 2021 (27)
**4**	+	–	+	+	–	–	–	3	Liu et al., 2022 (34)
**5**	+	+	–	+	–	–	–	3	Gambichler et al., 2021 (35)
**6**	+	–	+	–	–	–	–	2	Kasperkiewicz et al., 2010 (36)
**7**	+	+	–	–	–	–	–	2	Cho and Kim, 2003 (37)
**8**	+	–	+	–	–	–	–	2	Meijer et al., 2016 (38)
**9**	+	–	+	–	–	–	–	2	Goetze et al., 2017 (16)
**10**	+	–	–	–	+	–	–	2	Yamada et al., 2006 (28)
**11**	+	–	+	–	–	–	–	2	Yamane et al., 2007 (39)
**12**	+	+	–	–	–	–	–	2	Iwata et al., 2009 (40)
**13**	+	–	+	–	–	–	–	2	Goto-Ohguchi et al., 2009 (41)
**14**	+	–	+	–	–	–	–	2	Alloo et al., 2014 (42)

“+”, positive for indicated mucosal lesion; “-”, negative for indicated mucosal lesion.

Skin lesions were found in 51 of the 52 patients, however, only one patient was associated with psoriasis. In addition, there are two patients associated with malignancies, metastatic ovarian carcinoma and metastatic esophageal carcinoma (**Table 2**).

Histopathologically, 49 patients had pathological reports, which showed subepidermal blistering (98.0%), and inflammatory infiltrations of neutrophils (46.9%), eosinophils (38.8%) and lymphocytes (18.4%) (**Table 2**).

**Figure 1:**
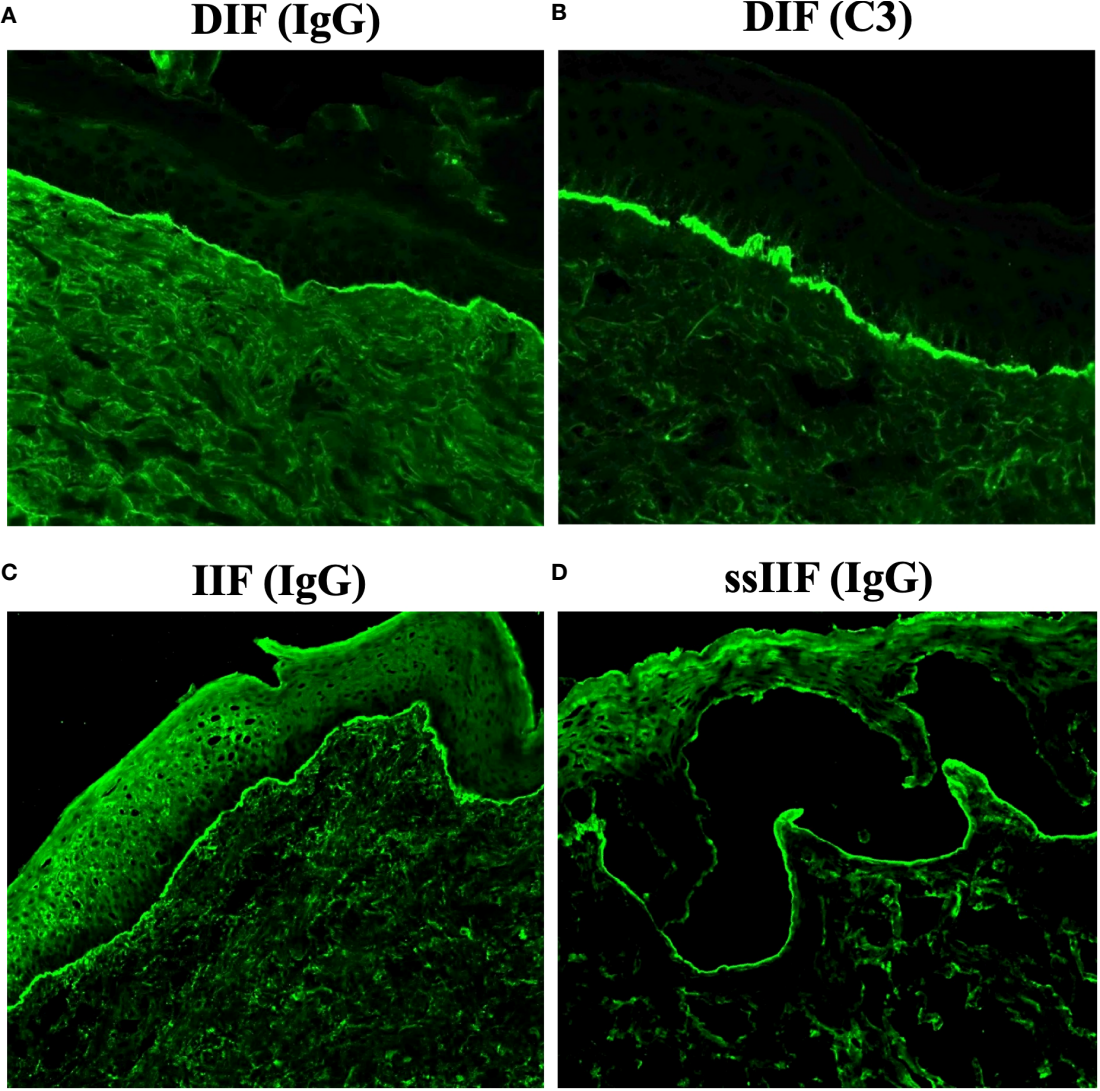
Representative results of various immunofluorescence tests for anti-p200 pemphigoid. **(A, B)** Direct immunofluorescence (DIF) for IgG **(A)** and C3 **(B)**. **(C)** Indirect immunofluorescence of normal human skin (IIF) for IgG. **(D)** Indirect immunofluorescence of 1M NaCl-split normal human skin (ssIIF) for IgG. All these representative results were originated from an anti-p200 pemphigoid patient with mucosal lesions.

Concerning the results of immunofluorescence tests, the positive rates of IgG (**Figure 1A**) and C3 (**Figure 1B**) depositions to BMZ in DIF were 94.4% and 97.1%, respectively, positive IgG reactivity rate in IIF of normal human skin (**Figure 1C**) was 80.0%, and positive IgG reactivity with dermal side in ssIIF (**Figure 1D**) was 97.2%. A few patients showed IgA and/or IgM autoantibodies in these immunofluorescence tests (**Table 2**). By IB of human dermal extract, the positive rates of IgG against the 200 kDa p200 were 97.8%. In addition, anti-p200 autoantibodies were also detected by IB of various LMγ1 recombinant proteins in some cases (**Table 2**).

IgG autoantibodies targeting non-p200 autoantigens were reported in 30.8% patients. The autoantibodies against additional antigens, LM332 in 9 cases, BP180 in 6 cases, Type VII collagen in 2 cases and BP230 in one case were also detected. Moreover, for IgG autoantibodies against LM332, LMγ2 subunit was the most frequently recognized, followed by LMβ3 and LMα3 subunits (**Table 2**).

Because of the lack of detailed information for the treatments in most cases, we did not analyze the treatments in the present study.

### The clinical, histopathological and immunological results of two subgroups of anti-p200 pemphigoid with mucosal lesions

According to the autoantigens detected, the 52 cases of anti-p200 pemphigoid with mucosal lesions were divided into two subgroups as sole p200 Ag-M (sole p200 antigen-anti-p200 pemphigoid with mucosal lesions) subgroup (36 cases), which reacted only with p200, and multiple antigens (Ags)-M (multiple antigens-anti-p200 pemphigoid with mucosal lesions) subgroup (16 cases), which reacted with p200 and other antigens. The detailed clinical, histopathological and immunological features of these two groups were summarized in **Table 2**.

Patients in sole p200 Ag-M and multiple Ags-M subgroups showed similar clinical, histopathological and immunological features, except that multiple Ags-M subgroup (25.0%) had higher ratio of nasal lesions than sole p200 Ag-M subgroup (2.8%) (p<0.05) (**Tables 4** and **5**).

**Table 4:** Comparison of clinical features between anti-p200 pemphigoid with mucosal lesions and other AIBD subtypes.

Item		Anti-p200 pemphigoid				Anti-LM332-type MMP (55)	Anti-BP180-type MMP (332)	EBA (105)
		With mucosal lesions (sole p200 Ag-M, 36)	With mucosal lesions (multiple Ags-M, 16)	With only skin lesions (sole p200 Ag-S, 82)	With only skin lesions (multiple Ags-S, 20)			
**Mucosal lesions**	Oral	27 in 36 (75.0%)	12 in 16 (75.0%)	/	/	50 in 55 (90.9%) *	284 in 332 (85.5%)	66 in 105 (62.9%)
	Ocular	3 in 36 (8.3%)	3 in 16 (18.8%)	/	/	25 in 55 (45.5%) *	97 in 332 (29.2%) *	3 in 105 (2.9%)
	Genital	8 in 36 (22.2%)	6 in 16 (37.5%)	/	/	6 in 55 (10.9%)	32 in 332 (9.6%) *	7 in 105 (6.7%) *
	Nasal	1 in 36 (2.8%)	4 in 16 (25.0%) *	/	/	1 in 55 (1.8%)	11 in 332 (3.3%)	1 in 105 (1.0%)
	Esophageal	0	1 in 16 (6.3%)	/	/	3 in 55 (5.5%)	14 in 332 (4.2%)	1 in 105 (1.0%)
	Pharyngeal	0	1 in 16 (6.3%)	/	/	11 in 55 (20.0%) *	42 in 332 (12.7%) *	Unknown
	Laryngeal	0	0	/	/	10 in 55 (18.2%) *	35 in 332 (10.5%)	Unknown
**Lesion on single mucosa**		29 in 36 (80.6%)	9 in 16 (56.2%)	/	/	Unknown	127 in 200 (63.5%) *	Unknown
**Lesion on multiple mucosa**		7 in 36 (19.4%)	7 in 16 (43.8%)	/	/	Unknown	Unknown	Unknown
**Skin lesions**		35 in 36 (97.2%)	16 in 16 (100.0%)	82 in 82 (100.0%)	20 in 20 (100.0%)	37 in 55 (67.3%) *	132 in 332 (39.8%) *	69 in 105 (65.7%) *
**Psoriasis**		0	1 in 16 (6.3%)	26 in 82 (31.7%) *	6 in 20 (30.0%) *	Unknown	Unknown	Unknown
**Malignancy**		1 in 36 (2.8%)	1 in 16 (6.3%)	1 in 82 (1.2%)	0 in 20 (0.0%)	8 in 55 (14.5%)	21 in 332 (6.3%)	7 in 105 (6.7%)

LM332, laminin 332; MMP, mucous membrane pemphigoid; EBA, epidermolysis bullosa acquisita; Ag, antigen; all 52 cases of anti-p200 pemphigoid with mucosal lesions were classified into 2 subgroups,(i) those with only p200 as autoantigen (sole p200 Ag-M subgroup) and (ii) those with p200 plus other autoantigens (multiple Ags-M subgroup); all 102 cases of anti-p200 pemphigoid with only skin lesions were also classified into 2 subgroups, (i) those with only p200 as autoantigen (sole p200 Ag-S subgroup) and (ii) those with p200 plus other autoantigens (multiple Ags-S subgroup); “/”, not applicable; *, p < 0.05 when compared with sole p200 Ag-M subgroup.

**Table 5:** Comparison of immunological features between anti-p200 pemphigoid with mucosal lesions and other AIBD subtypes.

Item		Anti-p200 pemphigoid				Anti-LM332-type MMP (55)	Anti-BP180-type MMP (332)	EBA (105)
		With mucosal lesions (sole p200 Ag-M, 36)	With mucosal lesions (multiple Ags-M, 16)	With only skin lesions (sole p200 Ag-S, 82)	With only skin lesions (multiple Ags-S, 20)			
DIF	BMZ (IgG)	23 in 24 (95.8%)	11 in 12 (91.7%)	17 in 17 (100.0%)	67 in 68 (98.6%)	55 in 55 (100.0%)	108 in 132 (81.8%)	80 in 83 (96.4%)
	BMZ (IgA)	5 in 7 (71.4%)	1 in 5 (20.0%)	1 in 5 (20.0%)	9 in 12 (75.0%)	Unknown	47 in 71 (66.2%)	9 in 15 (60.0%)
	BMZ (IgM)	0	1 in 4 (25.0%)	0	2 in 4 (50.0%)	Unknown	14 in 40 (35.0%)	Unknown
	BMZ (C3)	23 in 24 (95.8%)	11 in 11 (100.0%)	17 in 17 (100.0%)	70 in 70 (100.0%)	41 in 55 (74.5%)	110 in 127 (86.6%)	72 in 75 (96.0%)
IIF	BMZ (IgG)	4 in 5 (80.0%)	4 in 5 (80.0%)	8 in 8 (100.0%)	9 in 9 (100.0%)	23 in 55 (41.8%)	132 in 319 (41.4%)	96 in 105 (91.4%)
	BMZ (IgA)	1 in 1 (100.0%)	0	0	1 in 1 (100.0%)	Unknown	26 in 285 (9.1%)	3 in 48 (6.3%)
ssIIF	epidermal side (IgG)	0	0	0	1 in 1 (100.0%)	Unknown	207 in 317 (65.3%)	10 in 104 (9.6%)
	epidermal side (IgA)	0	0	1 in 1 (100.0%)	1 in 1 (100.0%)	Unknown	151 in 282 (53.55%)	9 in 45 (20.0%)
	both epidermal and dermal side (IgG)	0	4 in 4 (100.0%)	11 in 11 (100.0%)	0	Unknown	Unknown	10 in 104 (9.6%)
	both epidermal and dermal side (IgA)	0	0	0	1 in 1 (100.0%)	Unknown	Unknown	2 in 45 (4.4%)
	dermal side (IgG)	26 in 27 (96.3%)	9 in 9 (100.0%)	7 in 7 (100.0%)	58 in 58 (100.0%)	48 in 55 (87.3%)	62 in 317 (19.6%) *	93 in 104 (89.4%)
	dermal side (IgA)	4 in 5 (80.0%)	1 in 1 (100.0%)	0	2 in 3 (66.7%)	Unknown	4 in 282 (1.42%) *	3 in 45 (6.7%) *
IB	normal human epidermal extract (IgG)	6 in 9 (66.7%)	0	1 in 2 (50.0%)	3 in 4 (75.0%)	Unknown	Unknown	Unknown
	normal human dermal extract (IgG)	29 in 30 (96.7%)	15 in 15 (100.0%)	20 in 20 (100.0%)	57 in 57 (100.0%)	Unknown	Unknown	92 in 103 (89.3%)
	normal human dermal extract (IgA)	1 in 1 (100.0%)	0	0	1 in 2 (50.0%)	Unknown	Unknown	1 in 7 (14.3%)

LM332, laminin 332; MMP, mucous membrane pemphigoid; EBA, epidermolysis bullosa acquisita; Ag, antigen; all 52 cases of anti-p200 pemphigoid patients with mucosal lesions were classified into 2 subgroups, (i) those with only p200 as autoantigen (sole p200 Ag-M subgroup) and (ii) those with p200 plus other autoantigens (multiple Ags-M subgroup); all 102 cases of anti-p200 pemphigoid patients with only skin lesions were also classified into 2 subgroups, (i) those with only p200 as autoantigen (sole p200 Ag-S subgroup) and (ii) those with p200 plus other autoantigens (multiple Ags-S subgroup); DIF, direct immunofluorescence; BMZ, basement membrane zone; IIF, indirect immunofluorescence; ssIIF, IIF using 1M NaCl-split skin; IB, immunoblotting; *p < 0.05 when compared with sole p200 Ag-M subgroup.

### Comparative analyses of the data of sole p200 Ag-M subgroup with two more subgroups of anti-p200 pemphigoid with only skin lesions

In the present study, the sole p200 Ag-M subgroup is the most important group to explore the potent contribution of anti-p200 autoantibodies to mucosal lesions. Therefore, sole p200 Ag-M subgroup was next employed to compare with two more subgroups of anti-p200 pemphigoid with only skin lesions, designated as sole p200 Ag-S (sole p200 antigen-anti-p200 pemphigoid only with skin lesions) subgroup (82 cases), which reacted only with p200, and as multiple Ags-S subgroup (multiple antigens-anti-p200 pemphigoid only with skin lesions) (20 cases), which reacted with p200 and other antigens.

Except for mucosal lesions, the sole p200 Ag-M subgroup (0%) contained a significantly lower percentage of psoriasis than the sole p200 Ag-S subgroup (31.7%) and multiple Ags-S subgroup (30%) (both p<0.05) (**Tables 4** and **5**).

### Comparative analyses of the data of sole p200 Ag-M subgroup with two MMP groups and EBA group

The clinical and immunological features of anti-LM332-type MMP group (55 cases) (43), anti-BP180-type MMP group (332 cases) (44) and EBA group (105 cases) (45) were shown in **Tables 4** and **5**.

Compared with anti-LM332-type MMP group, sole p200 Ag-M subgroup had significantly lower ratios on oral, ocular, pharyngeal and laryngeal lesions (all *p*<0.05), and higher ratio on skin lesions (*p*<0.05).

Compared with anti-BP180-type MMP group, sole p200 Ag-M subgroup had significantly lower ratios on ocular and pharyngeal lesions (both *p*<0.05), and higher ratios on genital lesions, single mucosal site lesion and skin lesion (all *p*<0.05).

Compared with EBA group, sole p200 Ag-M subgroup had significantly higher ratios on genital and skin lesions (both *p*<0.05), and higher ratio for IgA reactivity to the dermal side on ssIIF (*p*<0.05).

## Discussion

In the present study, 52 cases of anti-p200 pemphigoid with mucosal lesions from the literature were investigated for the potential contribution of anti-p200 autoantibodies to mucosal lesions. In these 52 cases, oral and genital lesions were the two most frequent mucosal lesions, 26.9% cases had lesions on multiple mucosal sites, in which oral and genital mucosae were also the most frequently affected mucosal sites.

Based on the results of autoantigens detected, the 52 cases of anti-p200 pemphigoid with mucosal lesions were classified into two subgroups, sole p200 Ag-M (36 cases), and multiple Ags-M (16 cases), both of which were similar in terms of clinical, histopathological and immunological features, except for higher ratio of nasal lesions appeared in multiple Ags-M subgroup.

Except for mucosal lesions, sole p200 Ag-M subgroup also showed lower frequency of psoriasis, when compared with other two subgroups of anti-p200 pemphigoid with only skin lesions, i.e., sole p200 Ag-S subgroup (82 cases) and multiple Ags-S subgroup (20 cases).

To determine the possible differences in the contribution to develop mucosal lesions between autoantibodies against p200 and other known MMP autoantigens, we next compared sole p200 Ag-M subgroup with anti-LM332-type MMP and anti-BP180-type MMP groups. The results indicated that anti-p200 autoantibodies might induce less frequently oral, ocular, pharyngeal and laryngeal lesions compared with anti-LM332 autoantibodies, while anti-p200 autoantibodies might produce less frequently ocular and pharyngeal lesions but more frequently genital lesions and single mucosa lesion, when compared with anti-BP180 autoantibodies.

Sole p200 Ag-M subgroup were also compared with EBA group, because both p200 and type VII collagen (EBA autoantigen) were located in dermal side of 1M NaCl-split skin. The results indicated that anti-p200 autoantibodies might induce more frequently genital and skin lesions when compared with anti-type VII collagen autoantibodies.

Importantly, anti-LM332-type MMP was the most commonly associated AIBD in anti-p200 pemphigoid with mucosal lesions, which further emphasize the necessity of detection of anti-LM332 autoantibodies in anti-p200 pemphigoid with mucosal lesions.

The results of the present study suggest that, in addition to LM332 and BP180, p200 is an important autoantigen of MMP. Therefore, anti-p200 pemphigoid with extensive mucosal lesions could be considered as anti-p200 MMP. Anti-p200 pemphigoid and anti-p200 MMP show the same autoantigen but different clinical features, which is the similar situation for BP and anti-BP180-type MMP.

IB of dermal extract is usually not used for the diagnosis of MMP, because this assay was mainly used for detection of antibodies to EBA antigen (type VII collagen) and p200. However, the results in the present review suggested the importance of IB of dermal extract for serological diagnosis of MMP, because p200 is also one of autoantigens of MMP. We occasionally encounter MMP cases, which do not show autoantibodies against known MMP-related autoantigens, such as LM332, BP180 and integrin α6β4. In this situation, we would like to suggest detection of autoantibodies against p200.

This review was summarized and analyzed on the information from patients previously reported as case reports and case series. These published articles were not completely comprehensive, and some of them were only recorded with the clinical data and simple medications. Due to lack uniformity information, therapy profile was not analyzed. Additionally, 52 cases of anti-p200 pemphigoid with mucosal lesions in the literature were originated from only eight countries, which is possibly because very few countries have the abilities to diagnose this disease. Anti-BP180-type MMP group used in the present study is not a pure group with only anti-BP180 antibodies, which might influence the comparative results.

In summary, anti-p200 pemphigoid with mucosal lesions showed unique clinical features of higher frequency of genital lesions and very rare psoriasis, and distinct immunological features of high incidence of cooccurrence of autoantibodies against LM332. p200 is also a very important autoantigen of MMP. The epitopes and pathogenesis of anti-p200 autoantibodies were still inconclusive.

## Author contributions

XL and TH designed this project and revised this manuscript. YG and HQ performed data collection, analysis and prepare the manuscript. All authors contributed to the article and approved the submitted version.

## References

[1] Saschenbrecker S Karl I Komorowski L Probst C Dähnrich C Fechner K. Serological diagnosis of autoimmune bullous skin diseases. Front Immunol (2019) 10:1974. doi: 10.3389/fimmu.2019.01974 31552014 PMC6736620

[2] Zillikens D Kawahara Y Ishiko A Shimizu H Mayer J Rank CV. A novel subepidermal blistering disease with autoantibodies to a 200-kDa antigen of the basement membrane zone. J Invest Dermatol (1996) 106(6):1333–8. doi: 10.1111/1523-1747.ep12349283 8752680

[3] Chen KR Shimizu S Miyakawa S Ishiko A Shimizu H Hashimoto T. Coexistence of psoriasis and an unusual IgG-mediated subepidermal bullous dermatosis: Identification of a novel 200-kDa lower lamina lucida target antigen. Br J Dermatol (1996) 134(2):340–6. doi: 10.1111/j.1365-2133.1996.tb07625.x 8746353

[4] Kawahara Y Zillikens D Yancey KB Marinkovich MP Nie Z Hashimoto T. Subepidermal blistering disease with autoantibodies against a novel dermal 200-kDa antigen. J Dermatol Sci (2000) 23(2):93–102. doi: 10.1016/s0923-1811(99)00093-6 10808126

[5] Dainichi T Kurono S Ohyama B Ishii N Sanzen N Hayashi M. Anti-laminin gamma-1 pemphigoid. Proc Natl Acad Sci U.S.A. (2009) 106(8):2800–5. doi: 10.1073/pnas.0809230106 PMC2650346 19196964

[6] Dainichi T Koga H Tsuji T Ishii N Ohyama B Ueda A. From anti-P200 pemphigoid to anti-laminin Gamma1 pemphigoid. J Dermatol (2010) 37(3):231–8. doi: 10.1111/j.1346-8138.2009.00793.x 20507386

[7] Kridin K Ahmed AR. Anti-P200 pemphigoid: A systematic review. Front Immunol (2019) 10:2466. doi: 10.3389/fimmu.2019.02466 31695695 PMC6817563

[8] Xie YH Wang SH Li SZ Zuo YG. Coexistence of anti-P200 pemphigoid and psoriasis: A systematic review. Front Immunol (2022) 13:839094. doi: 10.3389/fimmu.2022.839094 35317170 PMC8934418

[9] Séméria L Lamiaux M Quinchon JF Modiano P. Anti-P200 pemphigoid mimicking erythema multiforme. JAAD Case Rep (2022) 21:157–9. doi: 10.1016/j.jdcr.2022.01.011 PMC8866842 35242971

[10] Salmhofer W Kawahara Y Soyer HP Kerl H Nishikawa T Hashimoto T. A subepidermal blistering disease with histopathological features of dermatitis herpetiformis and immunofluorescence characteristics of bullous pemphigoid: A novel subepidermal blistering disease or a variant of bullous pemphigoid? Br J Dermatol (1997) 137(4):599–604. doi: 10.1111/j.1365-2133.1997.tb03794.x 9390339

[11] Kaune KM Kasperkiewicz M Tams D Bergmann M Zutt M. Anti-P200/Anti-Laminin γ1 pemphigoid and BP180 NC16A/4575- positive mucous membrane pemphigoid : Late diagnosis in a patient with disease-related loss of vision and multiple previous surgical interventions. Hautarzt (2015) 66(1):60–4. doi: 10.1007/s00105-014-3529-1 25339385

[12] Pastar Z Rados J Lipozencic J Dobric I Marinovic B Ishii N. Case of concurrent epidermolysis bullosa acquisita and anti-P200 pemphigoid–how to treat it? Int J Dermatol (2007) 46(3):295–8. doi: 10.1111/j.1365-4632.2006.02969.x 17343589

[13] Goyal N Rao R Shenoi SD Pai S Kumar P Bhogal BS. Epidermolysis bullosa acquisita and anti-P200 pemphigoid as major subepidermal autoimmune bullous diseases diagnosed by floor binding on indirect immunofluorescence microscopy using human salt-split skin. Indian J Dermatol Venereol Leprol (2017) 83(5):550–5. doi: 10.4103/ijdvl.IJDVL_678_16 28749386

[14] Kuang W Qian H Zhang Q Li W Hashimoto T Zeng X. Case report: Mucous membrane pemphigoid with IgG and IgA anti-laminin γ1 antibodies and IgA anti-laminin α5 antibodies. Front Immunol (2022) 13:903174. doi: 10.3389/fimmu.2022.903174 35720393 PMC9198329

[15] Wozniak K Kowalewski C Hashimoto T Ishii N Glinska-Wielochowska M Schwartz RA. Penicillin-induced anti-P200 pemphigoid: An unusual morphology. Acta Derm Venereol (2006) 86(5):443–6. doi: 10.2340/00015555-0117 16955192

[16] Goetze S Dumke AK Zillikens D Hipler UC Elsner P. Anti-P200/Laminin g 1 pemphigoid associated with metastatic oesophageal cancer. J Eur Acad Dermatol Venereol (2017) 31(4):e219–e21. doi: 10.1111/jdv.13983 27684497

[17] Kawahara Y Matsuo Y Hashimoto T Nishikawa T. A case of unique subepidermal blistering disease with autoantibodies against a novel dermal 200-kD antigen. Dermatology (1998) 196(2):213–6. doi: 10.1159/000017901 9568410

[18] Arora S Shetty VM Rao CR Pai SB Rao R. Serration pattern analysis as a practical adjunct tool for categorization of subepidermal autoimmune blistering diseases. Indian J Dermatol Venereol Leprol (2021) 87(6):778–86. doi: 10.25259/ijdvl_1232_20 34491679

[19] Hopkins CR Ren V Grover R Cockerell C Hsu S. When bullous pemphigoid is not bullous pemphigoid: The importance of going beyond direct immunofluorescence. Cureus (2022) 14(2):e22201. doi: 10.7759/cureus.22201 35308677 PMC8925621

[20] Zhiliang L Xiaodong Z Peiying J Suying F Baoxi W. A case of refractory antilaminin γ1 pemphigoid successfully treated with dexamethasone and mycophenolate mofetil. Int J Dermatol (2015) 54(5):e194–6. doi: 10.1111/ijd.12763 25773159

[21] Rai R Anand JB Shanmugasekar C Arunprasath P Chaitra V Zillikens D. Anti-P200 pemphigoid-the most common floor binding subepidermal autoimmune bullous disease in a tertiary care center in south India. Indian J Dermatol Venereol Leprol (2021) 87(6):787–91. doi: 10.25259/ijdvl_79_20 34160166

[22] Holtsche MM Goletz S von Georg A van Beek N Hübner F Pigors M. Serologic characterization of anti-P200 pemphigoid: Epitope spreading as a common phenomenon. J Am Acad Dermatol (2021) 84(4):1155–7. doi: 10.1016/j.jaad.2020.07.076 32711089

[23] Raffin D Delaplace M Roussel A Estève E. Anti-P200 pemphigoid: Remission under mycophenolate mofetil (Cellcept®). Ann Dermatol Venereol (2013) 140(12):784–7. doi: 10.1016/j.annder.2013.07.005 24315224

[24] Wald A Schmidt E Toberer F Gutschalk A Rentzsch K Enk AH. Overlap of bullous, anti-Laminin-332, and anti-P200 pemphigoid with concomitant anti-Contactin-1-Positive inflammatory polyneuropathy treated with intravenous immunoglobulins as a manifestation of epitope spreading. JAMA Dermatol (2019) 155(5):631–3. doi: 10.1001/jamadermatol.2018.5536 30892575

[25] Commin MH Schmidt E Duvert-Lehembre S Lasek A Morice C Estival JL. Clinical and immunological features and outcome of anti-P200 pemphigoid. Br J Dermatol (2016) 175(4):776–81. doi: 10.1111/bjd.14629 27037896

[26] Mitate E Kawano S Nakao Y Goto Y Kobayashi I Onozawa K. Concurrence of autoantibodies to both laminin γ1 and γ2 subunits in a patient with kidney rejection response. Acta Derm Venereol (2013) 93(1):114–5. doi: 10.2340/00015555-1395 22735827

[27] Sarrazin M Jouen F Duvert-Lehembre S. Refractory bullous pemphigoid with IgE anti-BP230 and IgG anti-P200 antibodies successfully treated with omalizumab. Ann Dermatol Venereol (2021) 148(1):60–2. doi: 10.1016/j.annder.2020.08.053 33478824

[28] Yamada T Suzuki M Koike Y Kida K Murata S Ishii N. A case of epidermolysis bullosa acquisita with autoantibody to anti-P200 pemphigoid antigen and exfoliative esophagitis. Dermatology (2006) 212(4):381–4. doi: 10.1159/000092292 16707891

[29] Vafia K Groth S Beckmann T Hirose M Dworschak J Recke A. Pathogenicity of autoantibodies in anti-P200 pemphigoid. PloS One (2012) 7(7):e41769. doi: 10.1371/journal.pone.0041769 22911854 PMC3404064

[30] Koga H Ishii N Dainichi T Tsuruta D Hamada T Ohata C. An attempt to develop mouse model for anti-laminin γ1 pemphigoid. J Dermatol Sci (2013) 70(2):108–15. doi: 10.1016/j.jdermsci.2013.01.001 23410740

[31] Cozzani E Di Zenzo G Calabresi V Carrozzo M Burlando M Longanesi L. Autoantibody profile of a cohort of 78 Italian patients with mucous membrane pemphigoid: Correlation between reactivity profile and clinical involvement. Acta Derm Venereol (2016) 96(6):768–73. doi: 10.2340/00015555-2311 26631393

[32] Li X Qian H Ishii N Yamaya M Fukuda H Mukai H. A case of concurrent antilaminin γ1 pemphigoid and antilaminin-332-Type mucous membrane pemphigoid. Br J Dermatol (2014) 171(5):1257–9. doi: 10.1111/bjd.13107 25262782

[33] Mitsuya J Hara H Ito K Ishii N Hashimoto T Terui T. Metastatic ovarian carcinoma-associated subepidermal blistering disease with autoantibodies to both the P200 dermal antigen and the gamma 2 subunit of laminin 5 showing unusual clinical features. Br J Dermatol (2008) 158(6):1354–7. doi: 10.1111/j.1365-2133.2008.08483.x 18294311

[34] Liu W Sun X Gao Y Li H Shi L Cheng L. A Chinese case of concurrent anti-laminin γ1 pemphigoid and anti-laminin 332-type mucous membrane pemphigoid. J Dermatol (2022) 00:1–3. doi: 10.1111/1346-8138.16513 35811504

[35] Gambichler T Richards C Bakirtzi M Susok L. Anti-laminin γ1 (P200) pemphigoid appearing with rosette-like herpetiform lesions. J Dermatol (2021) 48(1):e33–e4. doi: 10.1111/1346-8138.15663 33150639

[36] Kasperkiewicz M Hoppe U Zillikens D Schmidt E. Relapse-associated autoantibodies to BP180 in a patient with anti-P200 pemphigoid. Clin Exp Dermatol (2010) 35(6):614–7. doi: 10.1111/j.1365-2230.2009.03731.x 19874345

[37] Cho SB Kim SC. A Korean case of anti-P200 pemphigoid. Yonsei Med J (2003) 44(5):931–4. doi: 10.3349/ymj.2003.44.5.931 14584115

[38] Meijer JM Diercks GF Schmidt E Pas HH Jonkman MF. Laboratory diagnosis and clinical profile of anti-P200 pemphigoid. JAMA Dermatol (2016) 152(8):897–904. doi: 10.1001/jamadermatol.2016.1099 27167149

[39] Yamane N Sawamura D Nishie W Abe M Kodama K Adachi K. Anti-P200 pemphigoid in a 17-Year-Old girl successfully treated with systemic corticosteroid and dapsone. Br J Dermatol (2007) 156(5):1075–8. doi: 10.1111/j.1365-2133.2007.07810.x 17381449

[40] Iwata H Hiramitsu Y Aoyama Y Kitajima Y. A case of anti-P200 pemphigoid: Evidence for a different pathway in neutrophil recruitment compared with bullous pemphigoid. Br J Dermatol (2009) 160(2):462–4. doi: 10.1111/j.1365-2133.2008.08965.x 19077077

[41] Goto-Ohguchi Y Nishie W Akiyama M Tateishi Y Aoyagi S Tsuji-Abe Y. A severe and refractory case of anti-P200 pemphigoid resulting in multiple skin ulcers and scar formation. Dermatology (2009) 218(3):265–71. doi: 10.1159/000182268 19060472

[42] Alloo A Strazzula L Rothschild B Hawryluk E Levine D Hoang MP. Refractory antilaminin γ1 pemphigoid successfully treated with intravenous immunoglobulin and mycophenolate mofetil. J Eur Acad Dermatol Venereol (2014) 28(10):1401–3. doi: 10.1111/jdv.12352 24397852

[43] Li X Qian H Natsuaki Y Koga H Kawakami T Tateishi C. Clinical and immunological findings in 55 patients with anti-laminin 332-type mucous membrane pemphigoid. Br J Dermatol (2021) 185(2):449–51. doi: 10.1111/bjd.20099 33811327

[44] Yasukochi A Teye K Ishii N Hashimoto T. Clinical and immunological studies of 332 Japanese patients tentatively diagnosed as anti-BP180-Type mucous membrane pemphigoid: A novel BP180 c-terminal domain enzyme-linked immunosorbent assay. Acta Derm Venereol (2016) 96(6):762–7. doi: 10.2340/00015555-2407 26984589

[45] Hashimoto T Jin Z Ishii N. Clinical and immunological studies for 105 Japanese seropositive patients of epidermolysis bullosa acquisita examined at kurume university. Expert Rev Clin Immunol (2016) 12(8):895–902. doi: 10.1080/1744666x.2016.1196136 27247994

